# Reduced lifetime fitness (growth, body condition and survivability) of hatchery‐reared tiger pufferfish *Takifugu rubripes* compared to wild counterparts

**DOI:** 10.1111/jfb.15199

**Published:** 2022-09-07

**Authors:** Yoshimi Ogino, Atsuko Yamaguchi

**Affiliations:** ^1^ Graduate School of Fisheries and Environmental Sciences Nagasaki University Nagasaki Japan

**Keywords:** age and growth, Ariake Bay, East China Sea, fisheries enhancement, life history, otolith section, vertebral section

## Abstract

Tiger pufferfish *Takifugu rubripes* (order Tetraodontiformes, family Tetraodontidae) is a highly exploited species and stocks continue to decline, although hatchery‐reared juveniles have been released since 1965 for stock enhancement. To determine why the stock has not recovered through hatchery‐release practices, this study investigated and compared the population characteristics of wild and hatchery‐origin fish. The length–mass relationship showed that hatchery‐origin fish were skinnier, with males weighing less than 90% of the mass of wild males of the same length. The hepatosomatic index tended to be lower in hatchery‐origin fish. Age was estimated using the otolith‐based method, and the estimates were more accurate and precise than those obtained by the conventional vertebra‐based method. At the age of 2.9 years, an age at which specimens were the most abundant in catches, hatchery‐origin males weighed only 67% of wild males. The maximum observed age was 12 years for wild fish and 5 years for hatchery‐origin fish. The instantaneous total mortality rates of hatchery‐origin fish were more than twice as high as those of wild fish. In summary, the hatchery‐origin fish had poor health status, poor growth and high mortality, and their fitness in natural environments was therefore hypothesized to be low throughout life.

## 1 INTRODUCTION

The global exploitation rates of marine fish are estimated to be below long‐term sustainable levels at the whole ecosystem level, but some species with high economic value are overexploited as a result of intensive and selective fishing (Zhou *et al*., 2015). Enhancement of such fisheries through releases of hatchery‐reared juveniles has been proposed and in some cases implemented for more than 100 years, but the effectiveness of this approach remains controversial (Hilborn, 1998; Svåsand *et al*., 2000; Travis *et al*., 1998). Currently, more than 180 marine species, including salmonids, are released annually by 20 countries, with Japan having released the greatest number of species (72) (Kitada, 2018, 2020). However, when considering cost‐effectiveness (hatchery‐rearing costs and contribution to fisheries resources), most projects are not profitable (Kitada, 2018, 2020). Furthermore, the negative effects of hatchery releases, such as a reduction in genetic diversity, have become increasingly apparent (Araki & Schmid, 2010). Additionally, hatchery‐origin individuals tend to differ from wild individuals in a wide range of morphological, behavioural, physiological and ecological attributes, and are of lower fitness than wild individuals in natural environments (Lorenzen *et al*., 2012).

**FIGURE 1:**
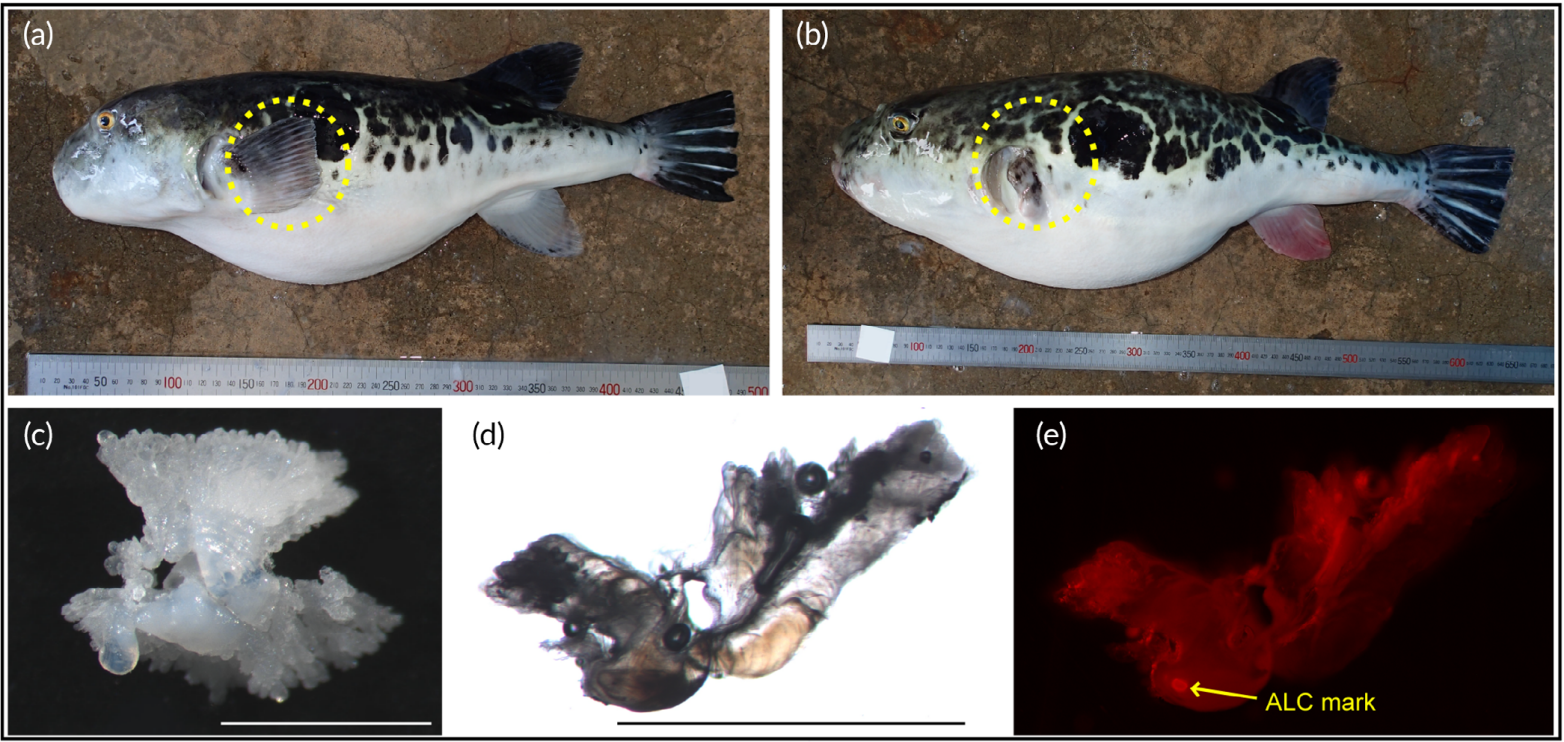
Identification of hatchery‐origin *Takifugu rubripes*. (a) A 451 mm total length (*L*_T_) wild male and (b) a 580 mm *L*_T_ hatchery‐origin female with left pectoral fin‐clipping. (c) Aberrant right sagittal otolith from a 453 mm *L*_T_ hatchery‐origin male and a section of the otolith viewed with transmitted light (d) and under green light (e). ALC, alizarin complexone. Scale bars: 1 mm

The tiger pufferfish *Takifugu rubripes* (Temminck & Schlegel, 1850) (Figure 1a) fishery is one of the fisheries for which hatchery releases are conducted with the aim of counteracting the serious decline of the stock. This species is found from the East China Sea to the Sea of Japan and the north‐west Pacific Ocean and is the largest pufferfish in the genus *Takifugu*, weighing up to 10 kg (Matsuura, 1997). Many species of pufferfish, including *T. rubripes*, possess the potent neurotoxin tetrodotoxin in the liver, ovary and, in exceptional species, muscle (Noguchi *et al*., 2006). For this reason, some countries have banned the trade of pufferfishes (Panão *et al*., 2016). However, Japan has a long tradition of eating pufferfishes (Kuo, 2019) and the fillets, once removed properly from the toxic parts, are exported to other countries, such as the USA and Singapore. In recent years, some countries have reconsidered the use of pufferfishes. This includes China, where consumption of certain pufferfishes was legalized in 2016 and the use of pufferfishes may be developed globally in future. Of all the pufferfishes, *T. rubripes* is the most economically valuable. It is an important resource because it is very tasty, with elastic white flesh, unlike any other fishes. At times when its market value is high, such as the beginning of the fishing season or the end of the year, the fish market price for wild *T. rubripes* often reaches 20,000 yen (US $200) per kilogram (Hamada, 1994). Therefore, *T. rubripes* stock is under high fishing pressure and has been overfished since 1992 (Uchida, 1994), with biomass in 2019 decreasing to approximately half of that in 2002, when stock assessment was started (FRA, 2020).

**FIGURE 2:**
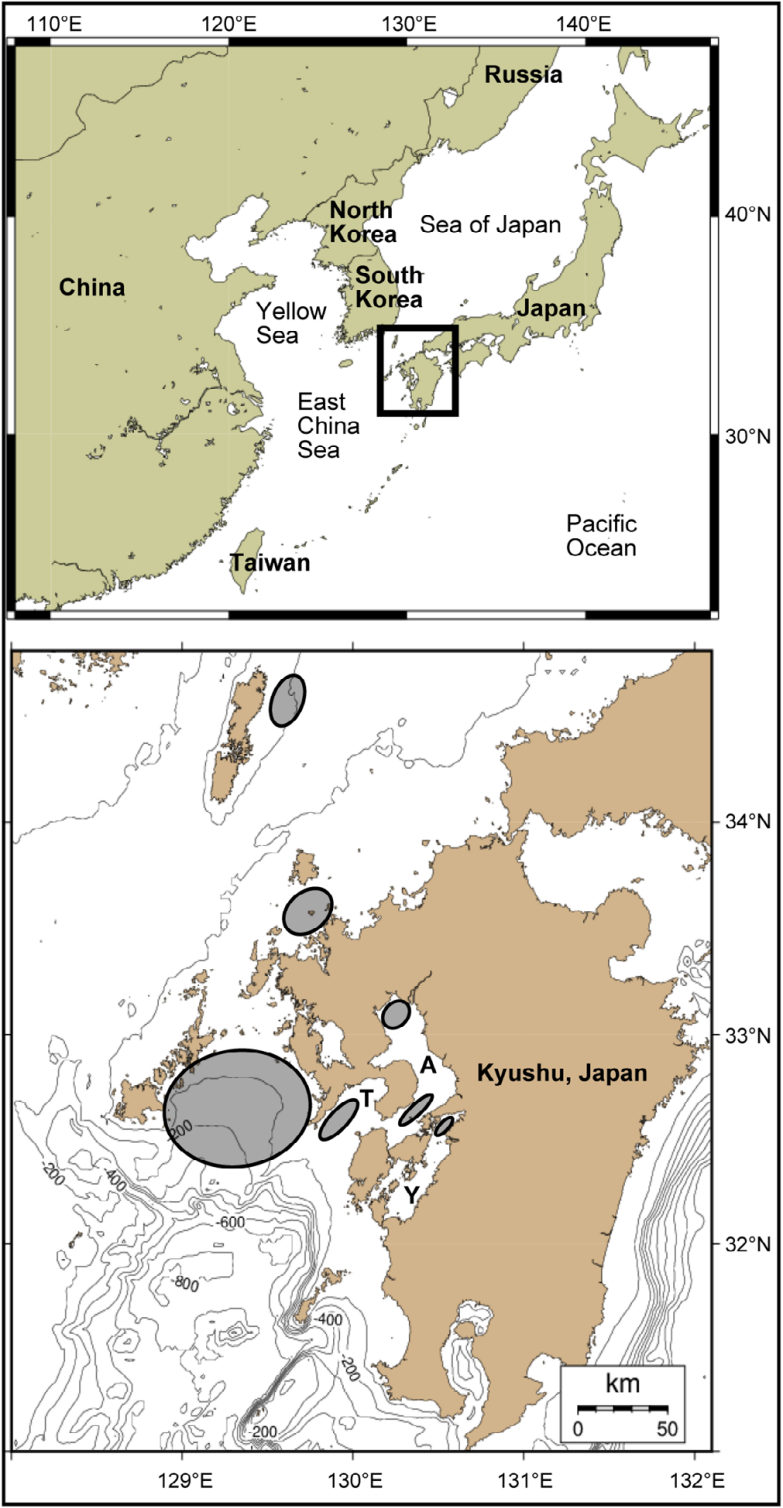
Location and sampling areas (indicated as shaded areas) of *Takifugu rubripes* examined from 2011 to 2020. A, Ariake Bay; T, Tachibana Bay; Y, Yatsushiro Bay

Harvest management is required to restore *T. rubripes* stocks, but this has not been successful to date (Kawata, 2017). One reason for this is that *T. rubripes* migrate over a relatively large area (Kawata, 2017). According to Yamada *et al*. (2007), who summarized previous ecological studies, the major stock of this species migrates throughout the East China Sea, Yellow Sea and Sea of Japan (Figure 2). Although the detailed migration routes are unknown, it is known that they gather to spawn at several specific sites (*e.g*., the mouths of bays) in spring and early summer. The larvae are carried by the tide to the inner part of bays (Yamaguchi & Kume, 2008). They spread through the bay as they grow and subsequently leave the bay in winter (Takita & Intong, 1991). At each stage of the life history, fishermen catch this species in specific places and seasons using specific methods. This makes it difficult to build consensus for fair catch limits, and to date there are no unified harvest management measures (Kawata, 2017). Against this background, the hatchery‐release programmes have been operated by the Japanese government for the purpose of stock enhancement and/or restoration since 1965 (Iwamoto & Fujimoto, 1997). As a result, studies of *T. rubripes* have concentrated on hatchery‐release techniques, such as improving recapture rates rather than on understanding the stock dynamics of wild fish. For example, in the Nagasaki area of Ariake Bay, there is a system whereby many hatchery‐origin fish caught by commercial fishermen are secured by Nagasaki Prefectural Institute Fisheries, and they have reported high recapture rates of hatchery‐origin fish and high benefit rates of the hatchery‐release programmes (Matsumura, 2005). However, population dynamics modelling shows that, if overfishing persists, releasing hatchery fish can enhance the fishery but will tend to further reduce, rather than restore, the wild population (Lorenzen 2005). The nursery grounds of wild *T. rubripes* are extensive sandy‐mud shallow waters, such as the inner parts of bays, and only a dozen sites are known (Matsuura, 1997; Yamada *et al*., 2007). In the current hatchery‐release programme, it is recommended to release large hatchery‐reared juveniles (70 mm total length) into these nursery grounds to increase their survival rate (FRA, 2020; Matsumura, 2005). For species with restricted nursery grounds, the release of hatchery‐reared juveniles into these grounds will elicit compensatory density‐dependent mortality, resulting in the ‘replacement’ of wild juveniles by the hatchery‐reared juveniles, as shown in some studies (Hilborn & Eggers, 2000; Kitada & Kishino, 2006; Sweeting *et al*., 2003). The magnitude of this replacement may vary from virtually none (a complete addition to the stock) to a complete replacement (no addition to the stock) and will typically be intermediate (Lorenzen *et al*., 2012). Unfortunately, in hatchery‐release programmes for *T. rubripes*, hatchery‐origin fish that have survived until fished are considered to be completely added to the stock (replacement with wild fish is none), without any evidence or investigation (FRA, 2020; Matsumura, 2005). In addition, although appropriate genetic management plans are essential for responsible hatchery‐release programmes (Grant *et al*., 2017), such plans have not yet been considered in the case of *T. rubripes*. In recent years, 1–2 million hatchery‐reared juveniles have been released annually, and the proportion of hatchery‐origin fish in landed age‐0 fish has reached 20%–30% (FRA, 2020). However, the decreasing trend in *T. rubripes* stocks continues, and the question remains: are hatchery‐release programmes really effective in restoring stocks?

To assess the true effect of hatchery‐release programmes, it is necessary to study various aspects of hatchery‐origin fish throughout their lives in natural environments and compare these characteristics with wild fish. However, only a few such studies have been conducted on *T. rubripes*. For example, although the gonadosomatic index, an indicator of maturity status, has been compared between wild and hatchery‐origin *T. rubripes* (Matsumura, 2006), neither the hepatosomatic index nor the length–mass relationship, indicators of health status, have been compared. Feeding habits and migration have also not been investigated. However, growth comparisons between wild and hatchery‐origin *T. rubripes* have been conducted by Ueta *et al*. (2010) based on vertebrae. Unfortunately, this is not the most exact method because age estimation by vertebrae is inferior to sectioned otoliths in terms of both accuracy and precision (Miller *et al*., 2010; VanderKooy *et al*., 2020). Although many age and growth studies have been conducted on *T. rubripes* because of their importance as a fishery resource (Matsuura, 1997; Yamada *et al*. 2007), their otoliths have never been used for age estimation. An exception was Matsumura (2006), who used otoliths to identify the year of release by checking the patterns of fluorescent staining and estimated age by the number of years elapsed until recapture, but this was only for hatchery‐released fish. No age estimation using the naturally formed seasonal increments on otoliths was conducted, including the more important wild fish, for *T. rubripes*. The reason for this is that both reading seasonal increments on whole otoliths and making them sections is difficult due to their very small size and complex shape, thus it has been considered impossible to use them for age estimation (Kotani *et al*., 1987; Ogushi, 1987; Ueta *et al*., 2010). This is a common problem in pufferfishes (AbouelFadl & Farrag, 2021; Başusta *et al*., 2020) and consequently age estimation using seasonal increments on sectioned otoliths has not been performed for species of this family (Tetraodontidae). However, this is a technical problem in the handling of otoliths and making otolith sections of *T. rubripes* should be possible with careful work.

First, the present study estimated the population parameters of *T. rubripes* more accurately than previous studies by using age estimates based on otolith sections. Concurrently, conventional vertebral age estimation was conducted and the results were compared. Second, the health status, growth and mortality rates of wild and hatchery‐origin fish were compared. If the hypothesis that the fitness of hatchery‐origin fish in the natural environment is low throughout their lives can be tested, it is hoped that this will provide important insights for reconsidering future hatchery‐release programmes, including *T. rubripes*, and for appropriate stock management.

## 2 MATERIALS AND METHODS

### 2.1 Fish collection and measurements

Between April 2011 and May 2020, 504 specimens of *T. rubripes* were collected from the catch of commercial fishermen using set nets, gill nets, purse seines and line fishing off the north‐west coast of Kyushu, Japan (Figure 2): mouth and inner part of Ariake Bay (*n* = 260), Tachibana Bay (*n* = 109), inner part of Yatsushiro Bay (*n* = 78), and three areas of East China Sea (*n* = 57).

The hatchery‐reared juveniles released by the Japanese government were marked by pectoral fin clipping or otolith staining (Figure 1), therefore individuals with these markings were identified as hatchery‐origin fish, whereas other individuals were considered wild fish. The presence of alizarin complexone marks on otoliths was checked under green light using a fluorescence microscope (AZ100, Nikon, Tokyo, Japan) for both whole and sectioned otoliths. The percentage of individuals with at least one aberrant otolith was determined because they were often observed. In the field or laboratory, specimen total length (*L*_T_) was recorded to the nearest 1 mm and total mass (*M*_T_), gonad mass (*M*_G_) and liver mass (*M*_L_) were recorded to the nearest 0.001 g for smaller fish or 0.1 g for larger fish. Individuals were classified as female, male or unsexed based on macroscopic examination of the gonads. Length–mass relationships were analysed by fitting an allometric model: Y = *a*X^*b*^, where Y is *M*_T_ and X is *L*_T_. Parameters *a* and *b* were estimated using nonlinear least‐squares (NLS) regression analysis. All other NLS and least‐squares (LS) regression analyses were performed using KyPlot 6.0 (Kyenslab, Tokyo, Japan) (Yoshioka, 2002). To compare the length–mass relationships between sexes and origins (wild or hatchery‐origin), analysis of covariance (ANCOVA) was performed on the log‐transformed datasets. All other ANCOVA tests were performed using KyPlot 6.0 with a significance level of 0.05. The gonadosomatic index (*I*_G_) was calculated using the formula *I*_G_ = *M*_G_ *L*_T_^−3^ × 10^6^ to investigate the reproductive status (Yamaguchi *et al*., 2000). The hepatosomatic index (*I*_H_) was calculated using the formula *I*_H_ = *M*_L_ *L*_T_^−3^ × 10^6^ to investigate the health status (Koyachi & Hashimoto, 1977).

### 2.2 Preparation of otolith and vertebral sections

**FIGURE 3:**
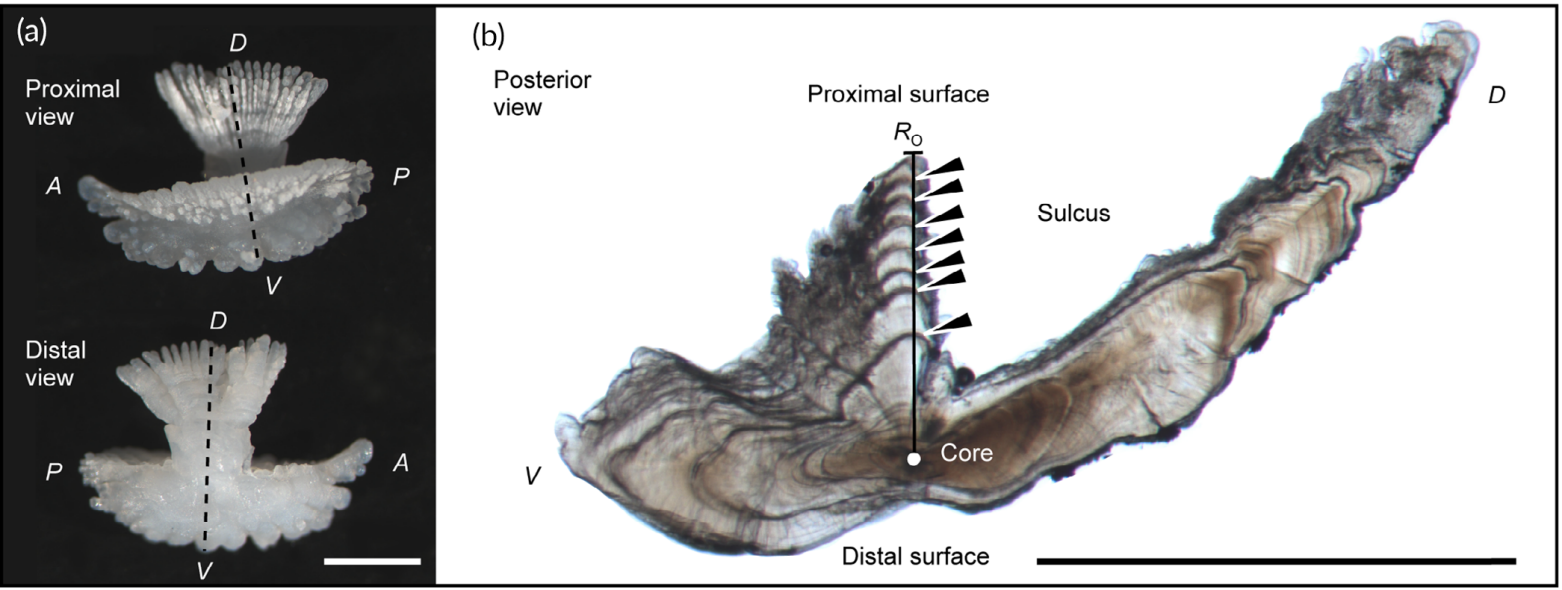
Whole (a) and sectioned (b) right sagittal otolith from a 568 mm total length wild male *Takifugu rubripes*. Broken lines indicate the sectioning plane. The otolith radius (*R*_O_) is the distance from the core to the edge of the ventral lobe next to the sulcus. Arrowheads indicate the opaque zones. A, anterior; P, posterior; D, dorsal; V, ventral. Scale bars: 1 mm

**FIGURE 4:**
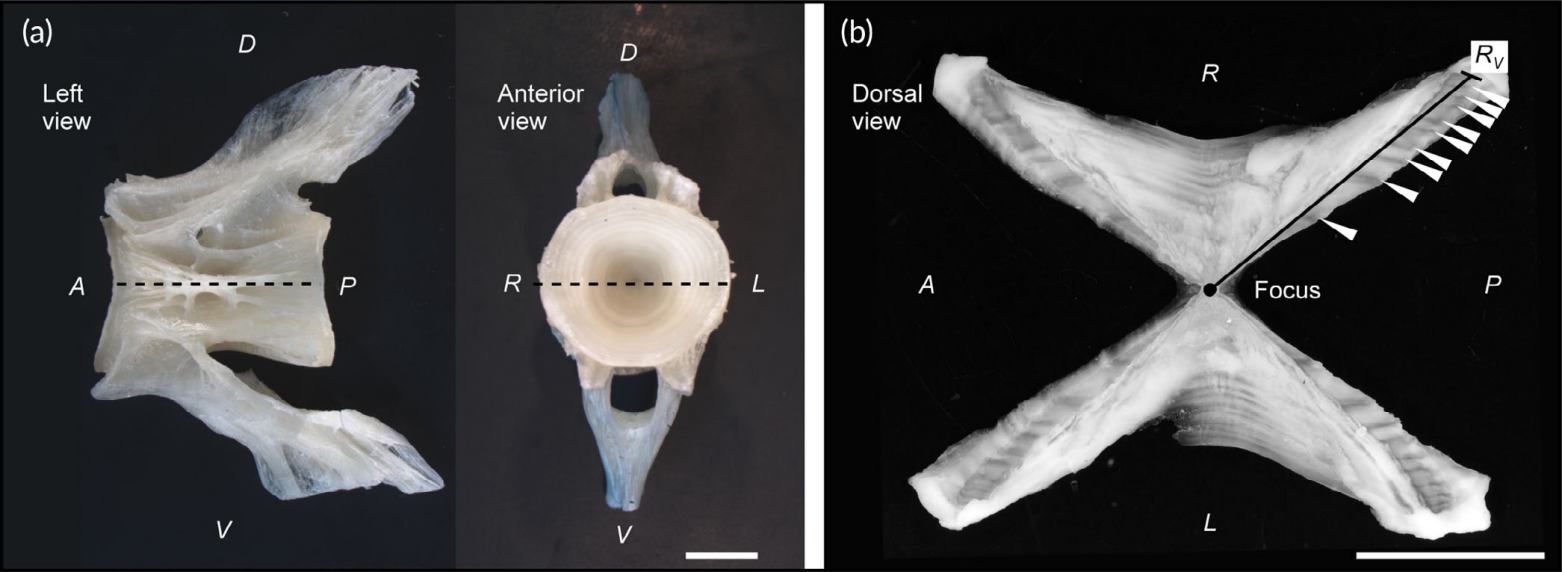
Whole (a) and sectioned (b) vertebral centrum from a 625 mm total length wild female *Takifugu rubripes*. Broken lines indicate the sectioning plane. The vertebral radius (*R*_V_) is the distance from the focus to the distal margin. Arrowheads indicate the translucent zones. A, anterior; P, posterior; D, dorsal; V, ventral; L, left; R; right. Scale bars: 5 mm

Sagittal otoliths were preserved dry after removal and the vertebrae were preserved in 70% ethanol after removal. Otolith and vertebral sections were prepared for 291 individuals where both otoliths and vertebrae were successfully preserved. Since the clarity of the zonation pattern on sectioned otoliths depends on the perpendicularity of the opaque zone to the sectioning surface (Jenke, 2002), otolith sections were carefully prepared according to the method described by Ogino *et al*. (2019). The resulting otolith sections were placed on glass slides and photographed by transmitted light using a digital camera (magnification 60×, DS‐Fi3; Nikon, Tokyo, Japan) mounted on a stereoscopic microscope (SMZ1270; Nikon). If the sectioning surface was not perfectly perpendicular to the opaque zone, the glass slides were tilted to improve the clarity of the opaque zone (VanderKooy, 2020). For vertebral sections, the 17th vertebra centrum was used following Ogushi (1987), and if sectioning of the 17th vertebra failed, the 16th or 18th vertebra was used. Each vertebral centrum was sectioned according to the method described by Kume *et al*. (2008), and the resulting section was immersed in glycerol and photographed under reflected light against a black background using a digital camera (magnification 0.5×) mounted on a stereoscopic microscope. On digital images, the opaque zone for the otolith sections (Figure 3) and the translucent zone for the vertebral sections (Figure 4) were counted because each was easy to read. The otolith radius (*R*_O_) was measured from the core to the ventral margin next to the sulcus (Figure 3), and the vertebral radius (*R*_V_) was measured from the focus to the margin along the corpus calcareum (Figure 4). The ANCOVA was performed on the log *R*_O_ and *L*_T_ datasets, and the *R*_V_ and *L*_T_ datasets to test differences between sexes and origins (wild or hatchery‐origin). The relationships between *L*_T_ and *R*_O_, and between *L*_T_ and *R*_V_ were expressed by three possible candidate models: a linear model, Y = *a* + *b*X; an exponential model, Y = *a*exp(*b*X); and the allometric model. Parameters *a* and *b* were estimated using NLS or LS regression analysis, then the best model for each dataset was selected using the small‐sample bias‐corrected Akaike's information criterion (AICc).

### 2.3 Precision and accuracy

The number of opaque zones for each otolith and translucent zones for each vertebra was counted three times by the same person, without knowledge of fish length or previous counts. To assess the precision of the opaque or translucent zone counts, the coefficient of variation (CV) was calculated using the three counts for each structure and averaged across fish to produce a mean CV (Chang, 1982). If the resulting counts were the same for two or more readings, the value was recorded as the number of opaque or translucent zones. If the counts differed in all three readings, the specimen was deemed illegible and excluded from the analyses. An age‐bias plot was used to investigate systematic variations in the number of opaque zones on the otolith and the number of translucent zones on the vertebra (Campana *et al*., 1995). In addition, the accuracy of the aging methods was investigated using known‐age fish (*n* = 16), which were collected from landings at the Tachibana Fisheries Cooperative Association in November 2019. These fish were hatchery reared to approximately 60–80 mm *L*_T_ and then cultured in offshore cages at Tachibana Bay (Figure 2) for 18 months (*n* = 10) or 30 months (*n* = 6).

### 2.4 Timing of annuli formation

The present study only investigated the formation cycle of the opaque zone on otoliths because previous studies have shown that the formation of the translucent zone of vertebrae is completed in May every year (Ogushi, 1987; Ueta *et al*., 2010). To investigate the timing of the formation of the first opaque zone, monthly changes in *R*_O_ and the presence of the first opaque zone were examined for small individuals (96 of the total specimens) caught in the nursery grounds (inner part of Ariake Bay and Yatsushiro Bay). Next, to investigate the timing of the formation of the second and subsequent opaque zones, monthly changes in the marginal increment (*i.e*., the distance between the outer edge of the single or outermost opaque zone and the otolith periphery) were analysed following the method described by Coulson *et al*. (2009).

### 2.5 Growth curves

In this study, sectioned otoliths were used for age estimation (see section 3.5). Based on the method of Fowler & Short (1998), each fish was assigned an age in months considering the periodicity of opaque zone formation, the date of capture and the universal birthdate of 1 April (*i.e*., the middle of the reproductive season; Takita & Intong, 1991). Growth curves were fitted to the length‐ and mass‐at‐age data for each sex and origin (wild or hatchery) using the NLS method. Following the multi‐model inference approach of Katsanevakis & Maravelias (2008), four growth models were used, namely the von Bertalanffy growth model (VBGM), the Gompertz model, the logistic model and the power model. Subsequently, the best model for each dataset was selected using the AICc. The VBGM was cubed when it was fitted to mass‐at‐age data, following Visconti *et al*. (2018). Using each selected best model, a likelihood ratio test was performed following the procedure of Cerrato (1990) to determine if growth could be modelled well by a single curve regardless of sex or origin (*α* = 0.05). In each test, data used were restricted so that the age range of the two groups was the same to ensure the validity of the comparisons (Haddon, 2011).

### 2.6 Mortality

To calculate the instantaneous total mortality rate (*Z*) from the age‐based catch curves, the two best methods of Smith *et al*. (2012) were used: the Chapman–Robson method and the weighted regression method. In this study, the age of maximum catch was used as the age of full recruitment because there was a major difference in catch between the age of maximum catch and 1 year older than the age of maximum catch. To assess the overall *Z* of each of the wild and hatchery‐origin populations, calculations were performed using combined sex data.

## 3 RESULTS

### 3.1 Identification of hatchery‐origin fish

Of the 504 *T. rubripes* caught, 97 were confirmed to have hatchery origin due to the presence of either fin clipping or otolith marking or both (Figure 1). Of the 48 fish for which otolith markings could be detected, 12 (25%) could not be detected on the surface of the whole otoliths, and markings could only be detected on otolith sections. The percentage of aberrant otoliths in wild *T. rubripes* was 4.6%, whereas that in hatchery‐released and hatchery‐cultured fish was 30.2% and 25.0%, respectively, which was more than six times higher than that in wild fish (Supporting Information Table S1). In some hatchery‐release operations, neither fin clipping nor otolith marking were conducted, but it was important in this study to distinguish between wild and hatchery‐origin fish as accurately as possible. Therefore, because the otolith abnormality rate is 0.6% for the closely related species *Takifugu xanthopterus* (Temminck & Schlegel 1850) (Y. Ogino & A. Yamaguchi, unpublished data), which live in the same area and whose life history is similar to that of the *T. rubripes*, an otolith abnormality rate of 4.6% for wild *T. rubripes* suggests that some of these fish are of hatchery origin. Thus, when comparing wild and hatchery‐origin fish in subsequent analyses, individuals without fin clipping or otolith markings, but with aberrant otoliths were excluded from the analysis.

### 3.2 Length–mass relationship

**FIGURE 5:**
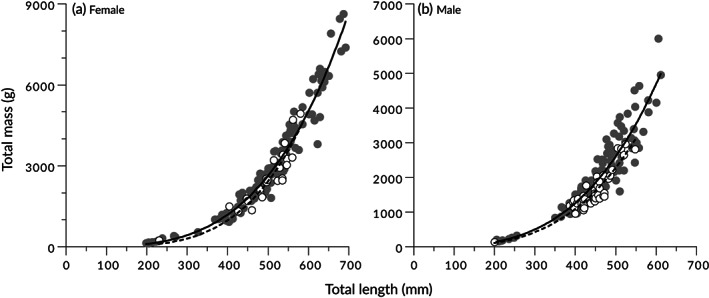
Length–mass relationships for (a) female and (b) male *Takifugu rubripes*. Wild fish: 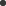, observed data; 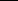, allometric model. Hatchery‐origin fish: 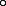, observed data; 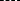, allometric model

Of the 504 *T. rubripes*, 188 were female, 226 were male and 90 were unsexed. ANCOVA with sex added as a covariate showed that *L*_T_–*M*_T_ relationships were significantly different between wild and hatchery‐origin fish (slopes, *F*_2, 403_ = 2.2, *P* = 0.1; intercepts, *F*_1, 405_ = 33, *P* < 0.001). Similarly, ANCOVA with origin added as a covariate showed that *L*_T_–*M*_T_ relationships were significantly different between females and males (slopes, *F*_2, 403_ = 2.7, *P* = 0.07; intercepts, *F*_1, 405_ = 4.5, *P* = 0.03), therefore the *L*_T_–*M*_T_ equation was calculated separately for each sex and origin (Table 1). A comparison of the calculated curves showed that hatchery‐origin fish tended to be skinnier (Figure 5), with the hatchery‐origin males weighing less than 90% of the mass of wild males of the same length.

**TABLE 1:** Allometric model parameters describing the relationships between total length (*L*_T_, mm) and total mass (*M*_T_, g) for *Takifugu rubripes*

	*M*_T_ = *aL*_T_^*b*^		
	*A*	*b*	*R* ^2^
	Females		
Wild	6.95 × 10^−7^ (3.50 × 10^−7^)	3.55 (0.08)	0.95
Hatchery‐origin	2.78 × 10^−8^ (7.55 × 10^−8^)	4.05 (0.43)	0.86
	Males		
Wild	4.77 × 10^−6^ (3.42 × 10^−6^)	3.24 (0.12)	0.86
Hatchery‐origin	2.81 × 10^−6^ (3.95 × 10^−6^)	3.30 (0.23)	0.83

*Note*: Values in parentheses are standard errors. *R* ^2^ = coefficient of determination.

### 3.3 Reproduction and body condition

**FIGURE 6:**
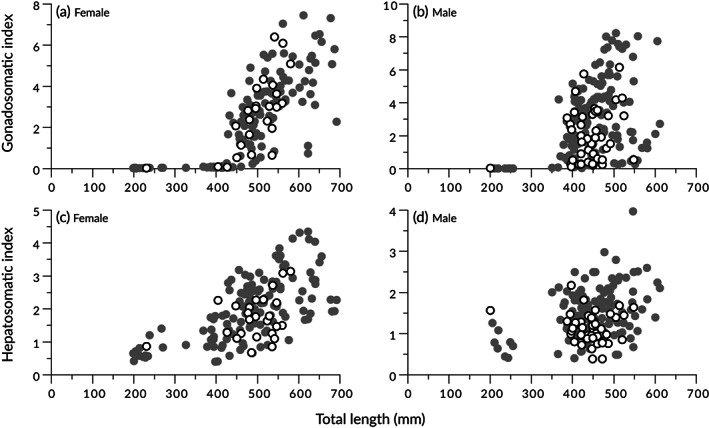
Relationships between total length to (a, b) gonadosomatic index and (c, d) hepatosomatic index for (a, c) female and (b, d) male *Takifugu rubripes* caught between February and May. 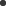, wild fish; 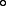, hatchery‐origin fish

The relationships between *L*_T_ and *I*_G_ or *I*_H_ were examined for *T. rubripes* (162 females and 194 males) caught during and around the spawning season (February–May). In females, the *I*_G_ tended to increase with length from approximately 430 mm (Figure 6a), while in males, the maximum *I*_G_ tended to increase with length from approximately 380 mm (Figure 6b). When the *I*_G_ was compared between wild and hatchery‐origin fish, no clear difference was observed in females (Figure 6a), but male hatchery‐origin fish tended to have a lower *I*_G_ than wild fish (Figure 6b). For *I*_H_, there was a trend of lower values in hatchery‐origin fish compared to those in wild fish for both sexes (Figure 6c,d). Furthermore, when the hatchery‐origin fish were divided into those with and without fin clipping and the mean *I*_H_ values were compared in a specific length range with a high number of individuals collected (females, 450–570 mm; males, 390–500 mm), the mean *I*_H_ values for both sexes were higher in wild fish, followed by hatchery‐origin fish without fin clipping and lastly hatchery‐origin fish with fin clipping (Table 2).

**TABLE 2:** Hepatosomatic index of *Takifugu rubripes* in a specific total length range (female 450–570 mm, male 390–500 mm)

	*n*	Total length (mm)				Hepatosomatic index			
		Min.	Max.	Mean	s.d.	Min.	Max.	Mean	s.d.
Female									
Wild	72	455	567	507	33	0.7	3.8	2.2	0.7
HnFC	12	450	561	509	32	1.1	3.1	1.9	0.6
HFC	7	460	560	523	36	0.7	2.7	1.4	0.7
Male									
Wild	99	390	498	440	31	0.4	3.0	1.4	0.5
HnFC	10	396	491	427	34	0.7	2.2	1.2	0.4
HFC	30	394	486	444	23	0.4	1.8	1.1	0.3

*Note*: HnFC, hatchery‐origin with no fin clipping; HFC, hatchery‐origin with fin clipping; s.d., standard deviation.

### 3.4 Growth of otoliths and vertebrae

**FIGURE 7:**
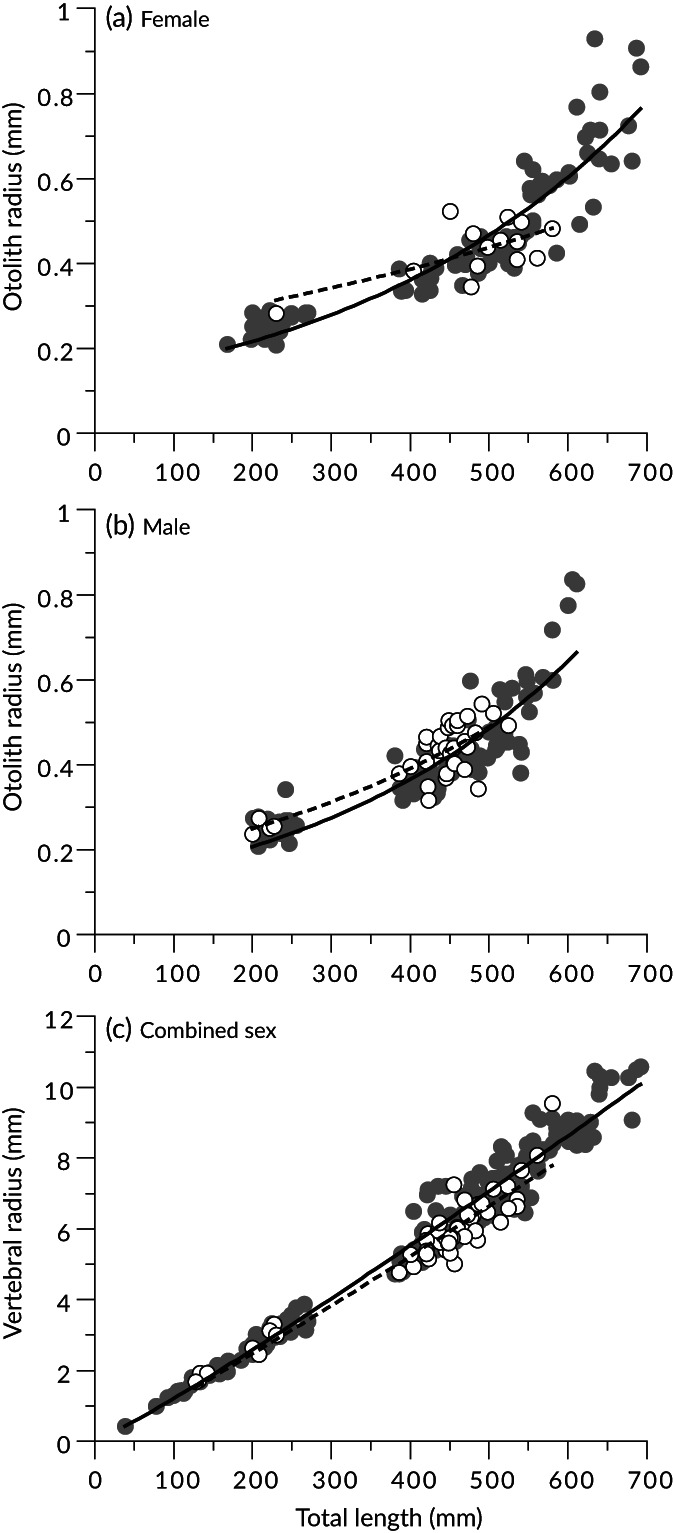
Relationships of (a, b) total length and otolith radius with exponential model and (c) total length and vertebral radius with allometric model for *Takifugu rubripes*. Wild fish: 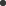, observed data; 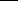, fitted model. Hatchery‐origin fish: 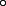, observed data; 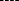, fitted model

The ANCOVA with sex added as a covariate showed significant differences by origin in both *L*_T_–*R*_O_ relationships (slopes, *F*_2, 240_ = 3.8, *P* = 0.02) and *L*_T_–*R*_V_ relationships (slopes, *F*_2, 240_ = 0.84, *P* = 0.4; intercepts, *F*_1, 242_ = 22, *P* < 0.001). The ANCOVA with origin added as a covariate showed significant differences by sex in the *L*_T_–*R*_O_ relationships (slopes, *F*_2, 240_ = 3.6, *P* = 0.03), but no significant differences in the *L*_T_–*R*_V_ relationships (slopes, *F*_2, 240_ = 2.3, *P* = 0.1; intercepts, *F*_1, 242_ = 0.17, *P* = 0.7). Therefore, the *L*_T_–*R*_O_ relationship was calculated separately for each sex and origin, while the *L*_T_–*R*_V_ relationship was calculated separately for each origin. The AICc comparisons showed that an exponential model was best fitted for the *L*_T_–*R*_O_ relationship of all the datasets except hatchery‐origin females, and the allometric and exponential models were best fitted for the *L*_T_–*R*_V_ relationship of wild and hatchery‐origin fish, respectively (Supporting Information Table S2). Therefore, the exponential model was used for all the *L*_T_–*R*_O_ relationships and the allometric model was used for all the *L*_T_–*R*_V_ relationships, enabling comparison across each dataset. The parameters of each equation are listed in Table 3. The *R*_O_ tends to be larger in hatchery‐origin fish than in wild fish (Figure 7a, b), whereas the *R*_V_ tends to be larger in wild fish (Figure 7c).

**TABLE 3:** Regression parameters describing the relationships between total length (*L*_T_, mm) and otolith radius (*R*_O_, mm), and *L*_T_ and vertebral radius (*R*_V_, mm) for *Takifugu rubripes*

	*R*_O_ = *a*exp(*bL*_T_)			*R*_V_ = *aL*_T_^*b*^		
	*a*	*b*	*R* ^2^	*a*	*b*	*R* ^2^
	Females			Combined sexes		
Wild	0.13 (0.01)	2.6 × 10^−3^ (0.1 × 10^−3^)	0.86	7.68 × 10^−3^ (0.85 × 10^−3^)	1.10 (0.02)	0.97
Hatchery‐origin	0.24 (0.06)	1.2 × 10^−3^ (0.5 × 10^−3^)	0.42	8.19 × 10^−3^ (3.25 × 10^−3^)	1.08 (0.06)	0.92
	Males					
Wild	0.12 (0.01)	2.8 × 10^−3^ (0.2 × 10^−3^)	0.81	–	–	–
Hatchery‐origin	0.16 (0.02)	2.2 × 10^−3^ (0.3 × 10^−3^)	0.65	–	–	–

*Note*: Values in parentheses are standard errors. *R* ^2^ = coefficient of determination.

### 3.5 Precision and accuracy

**FIGURE 8:**
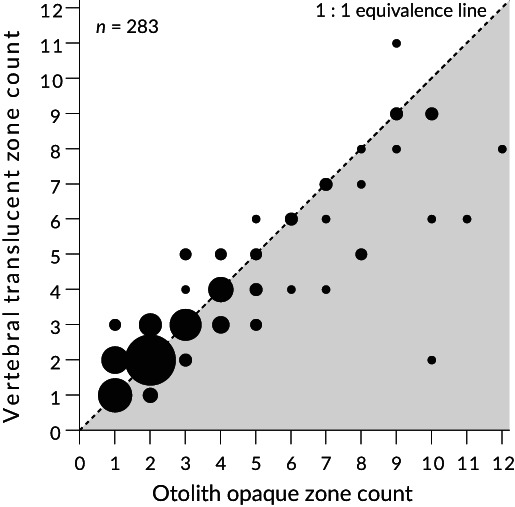
Comparisons of zone counts on otoliths and vertebrae from the same *Takifugu rubripes* individuals. The sizes of the circle are proportional to the number of individuals

The calculated mean CV was much lower for otoliths than for vertebrae, indicating that the precision of reading the opaque zone on otoliths was higher than of reading the translucent zone on vertebrae (Table 4). The proportion of individuals with differences in all three counts (illegible) was 0% for otoliths, compared with 2.7% for vertebrae. A comparison of the number of opaque zones on otoliths and translucent zones on vertebrae in the same individual showed that vertebrae tended to be counted less than otoliths at older ages (Figure 8). In cultured *T. rubripes* of known age, the number of opaque zones on all otoliths coincided with age, whereas the vertebrae of some individuals had more opaque zones than their actual age (Table 5 and Supporting Information Figure S1). Since otoliths were superior to vertebrae in terms of both precision and accuracy for age estimations, all subsequent age and growth analyses of *T. rubripes* were conducted using the sectioned otoliths.

**TABLE 4:** Percentage agreement of zone counts and mean coefficient of variation (CV) among three replicate readings for each aging structure from *Takifugu rubripes* (*n* = 291)

	Agreement (%) of zone counts			Mean CV (%)
	All readings Agree	Two readings Agree	All readings Disagree	
Otoliths	93.5	6.5	0.0	1.5
Vertebrae	74.9	22.3	2.7	9.1

**TABLE 5:** Annuli counts of otoliths and vertebrae from 1‐year‐old (*n* = 10) and 2‐year‐old (*n* = 6) cultured *Takifugu rubripes*

	Number of annuli				
	1	2	3	4	5
Otoliths					
1‐year‐old fish	10				
2‐year‐old fish		6			
Vertebrae					
1‐year‐old fish	9	1			
2‐year‐old fish		4		1	1

### 3.6 Timing of annuli formation

Monthly examination of the sectioned otoliths from juveniles showed that the first opaque zone was formed in May of the year following birth (Supporting Information Figure S2). In addition, the monthly trend of the marginal increment indicated that the second and later opaque zones formed in March–April (Supporting Information Figure S3). Due to the difficulty of collecting adult *T. rubripes*, the marginal increments from June to January could not be examined, but it was clear that the opaque zones on otoliths were formed annually from the examination of known age fish (Table 5). Therefore, it was concluded that the opaque zone on otoliths of *T. rubripes* completed its formation in March–May each year throughout the lifetime.

### 3.7 Growth curves

**FIGURE 9:**
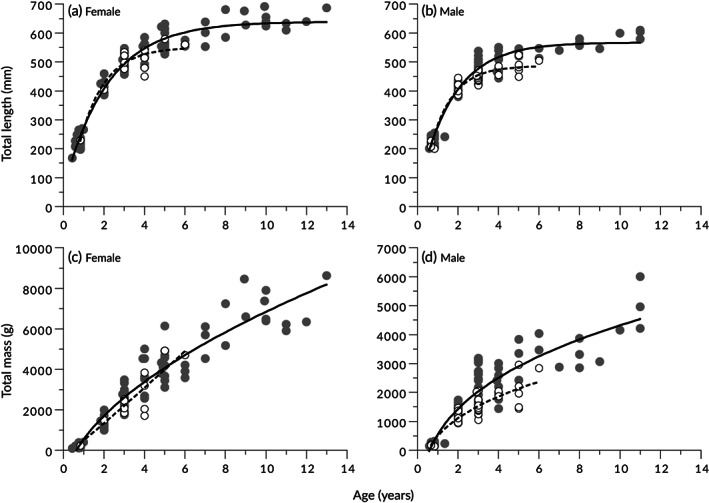
(a, b) Length‐at‐age data with von Bertalanffy growth model and (c, d) mass‐at‐age data with power model for (a, c) female and (b, d) male *Takifugu rubripes* based on ages estimated from otoliths. Wild fish: 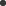, observed data; 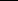, fitted model. Hatchery‐origin fish: 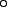, observed data; 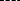, fitted model

As a result of the AICc comparisons, the VBGM and the power model were selected as the best growth models for *T. rubripes* length‐ and mass‐at‐age data, respectively (Supporting Information Table S3). When differences in growth curves according to sex or origin (wild or hatchery origin) were examined, significant differences were detected for all pairs except for those of wild *vs*. hatchery‐origin females (Table 6). The calculated growth curves and equations are shown in Figure 9 and Table 7, respectively. In both wild and hatchery‐origin fish, females tended to grow larger than males. At 2.9 years of age, the specimens which were most abundant in catches, the mean *M*_T_ was 2508 g [95% confidence interval (CI) = 2295–2720] for wild females (*n* = 22) and 2208 g (95% CI = 1960–2456) for wild males (*n* = 23). Growth differences between wild and hatchery‐origin fish were more pronounced in males, with a mean *M*_T_ at 2.9 years of age of hatchery‐origin males (*n* = 17) of 1476 g (95% CI = 1337–1616), which was only 67% that of wild males.

**TABLE 6:** Results of the likelihood ratio tests for the null hypothesis that growth of *Takifugu rubripes* could be modelled well, independent of sex and origin using a single curve

Data	Factor	*L*_T_–age		*M*_T_–age	
		χ^2^	*P*	χ^2^	*P*
Wild‐origin	Sex	41.2	<0.001	107	<0.001
Hatchery‐origin	Sex	14.0	0.003	20.9	<0.001
Female	Origin	2.09	0.6	1.33	0.7
Male	Origin	22.6	<0.001	21.4	<0.001

*Note*: The von Bertalanffy growth model and power model were used for the relationships between age and total length (*L*_T_), and age and total mass (*M*_T_), respectively. In all cases, d.f. = 3.

**TABLE 7:** Growth models fitted to length‐at‐age and mass‐at‐age data separately by sex and origin for *Takifugu rubripes*

	VBGM	Power model
Female		
Wild	*L*_*t*_ = 639 (1 − e^−0.46(*t* + 0.21)^)	*M*_*t*_ = −1950 + 2501 *t* ^0.546^
Hatchery‐origin	*L*_*t*_ = 551 (1 − e^−0.81(*t* − 0.17)^)	*M*_*t*_ = −267 + 769 *t* ^1.063^
Male		
Wild	*L*_*t*_ = 567 (1 − e^−0.58(*t* + 0.14)^)	*M*_*t*_ = −3311 + 3860 *t* ^0.296^
Hatchery‐origin	*L*_*t*_ = 486 (1 − e^−0.92(*t* − 0.06)^)	*M*_*t*_ = −2247 + 2742 *t* ^0.291^

*Note*: *L*_*t*_ and *M*_*t*_ is total length (mm) and total mass (g) at age *t*, respectively. VBGM, von Bertalanffy growth model.

### 3.8 Mortality

**FIGURE 10:**
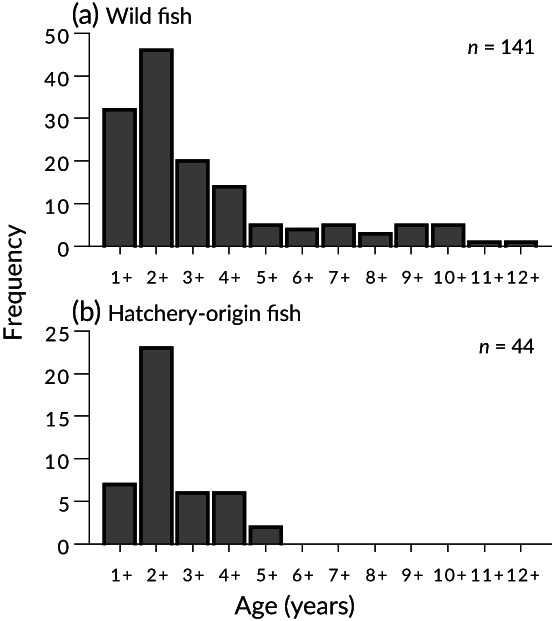
Age frequency histograms for (a) wild and (b) hatchery‐origin *Takifugu rubripes*

The maximum age of wild pufferfish was 12 years for females and 10 years for males, while hatchery‐origin fish only appeared up to 5 years of age for both sexes. For both wild and hatchery‐origin fish, age‐2 fish were the most abundant in the catch (Figure 10). The *Z* calculated by the Chapman‐Robson method was 0.40 for wild fish and 0.89 for hatchery‐origin fish, and that calculated by the weighted regression method was 0.35 for wild fish and 0.76 for hatchery‐origin fish. In both calculation methods, hatchery‐origin fish had more than twice the mortality of wild fish.

## 4 DISCUSSION

### 4.1 Age estimation of pufferfish

The present study is the first successful analysis of age and growth based on otolith sections of pufferfish (Tetraodontidae). Otoliths of pufferfishes are very small and complex in shape, making them difficult to section, and previous studies on age and growth have been performed using a length‐frequency method (Anju *et al*., 2019; Aydın, 2011; Pauly, 1991), observation of vertebrae (AbouelFadl & Farrag, 2021; Başusta *et al*., 2020; Farrag *et al*., 2015), observation of the otolith surface (Habib, 1977; Potter *et al*., 1988) or measuring the otolith size (Imai & Nonaka, 2015). The present study showed that the age estimates based on otolith sections were more accurate and precise than those obtained by the conventional vertebra‐based method. In addition, the mean CV of the vertebra‐based method of 9.1% exceeded the common acceptable level of 5% (Campana, 2001). Similar to pufferfishes, age estimation using otoliths is also difficult for filefishes (Monacanthidae), which are of the same order Tetraodontiformes (El‐Ganainy & Sabra, 2008; Kim *et al*., 2016). However, a few studies have successfully estimated filefishes age using otolith sections and also emphasized the limitations of age estimation using vertebrae in terms of accuracy and precision (Miller *et al*., 2010; Visconti *et al*., 2018). Since errors and biases in age estimation may lead to incorrect stock assessment and disturb stock management (Reeves, 2003; Yule *et al*., 2008), age estimation should also be based on sectioned otoliths for pufferfishes.

### 4.2 Age and growth of wild fish

The VBGM of wild *T. rubripes* based on otoliths in this study differed from VBGMs based on vertebrae in previous studies, and furthermore it differed among the previous studies (Supporting Information Table S4 and Figure S4). This discrepancy may be due to differences in sampling methods, calculation methods or actual growth variability, but is most likely due to age assessment error (and/or bias) among the studies due to the difficulty of reading annuli on vertebrae. The results of the present study showed that *T. rubripes* growth at old age was asymptotic in terms of *L*_T_ (Figure 9a,b), but continuous in terms of *M*_T_ (Figure 9c,d). The fecundity of this species is proportional to *M*_T_ (Hamada, 1994), therefore older fish will have higher fecundity and greater contribution to reproduction. The age frequency histogram of wild fish (Figure 10a) indicates the removal of older age classes of fish from the population (age‐class truncation), which is found in heavily exploited populations (Stewart, 2011).

### 4.3 Fitness of hatchery‐origin fish in the natural environment

Although many experimental studies have been performed on *T. rubripes* hatchery‐reared juveniles (*e.g*., Iwamoto & Fujimoto, 1997; Sakurai & Hamada, 2017), the long‐term fitness of hatchery‐origin individuals in the natural environment has rarely been investigated. Experiments in a seminatural environment using a salt pond mesocosm confirmed that the *I*_H_ of juvenile hatcheries decreased until 15 days after release, but then stabilized at the same level as wild juveniles (Shimizu *et al*., 2007). However, the results of the present study showed that hatchery‐origin fish released into the natural environment had a lower *I*_H_ and were skinnier than wild fish a few years after release. In addition, although a previous study concludes that fin‐clipping does not affect the growth of *T. rubripes* based on a 300‐day rearing experiment (Matsumura, 2001), the results of the present study show that the *I*_H_ of hatchery‐origin fish is even lower for individuals with fin‐clipping than individuals without fin‐clipping in the natural environment. These data emphasize the necessity of long‐term follow‐up studies after release to correctly understand the status of hatchery‐origin fish released into the natural environment.

Age estimation using sectioned otoliths showed that hatchery‐origin *T. rubripes* had poorer growth than wild fish. Similar trends have been reported by Ueta *et al*. (2010), who have used vertebrae to estimate *T. rubripes* age, presuming that the poor growth of hatchery‐origin fish is caused by a temporary fall in growth rate immediately after release. If their presumption is correct, the difference in growth between wild and hatchery‐origin fish should decrease with age, but no such trend has been observed in the data from either Ueta *et al*. (2010) or the present study, therefore their presumptions may be incorrect. In the present study, the hatchery‐origin fish were less healthy and skinnier than the wild fish, suggesting that their feeding ability was lower throughout their lives, which contributed to their lower growth rates. Similarly, the consistently lower growth rate of hatchery‐origin individuals has been reported in *Acanthopagrus butcheri* (Munro 1949), but the cause of this remains unidentified (Cottingham *et al*., 2020; Potter *et al*., 2008). Matsumura (2006) has observed a maximum age of recaptured hatchery‐origin *T. rubripes* of 8 years, but the present study observed only 5 years. Here, the *Z* value of hatchery‐origin fish was twice as high as that of wild fish, suggesting that hatchery‐origin fish had higher mortality. While it is possible that this could be the result of higher fishing mortality of hatchery‐origin fish (Jonsson *et al*., 2003; Mezzera & Largiadèr, 2001), it is more reasonable to presume that the higher *Z* value of hatchery‐origin fish is the result of higher natural mortality (Jonsson *et al*., 2003; Lorenzen, 2006), given the poor health status of the hatchery‐origin pufferfish. These data suggest that the negative effects of growing up in an unnatural hatchery environment persist throughout life.

### 4.4 Anomalies in hatchery‐origin fish

In the present study, the incidence of aberrant otolith formation in hatchery‐origin *T. rubripes* was higher than that in wild fish. Furthermore, the other normal otoliths tended to be distorted in shape overall for hatchery‐origin fish. These factors, in addition to the growth difference, would result in a significant difference in the *L*_T_–*R*_O_ relationships between wild and hatchery‐origin fish. The phenomenon where hatchery‐origin fish have a higher rate of aberrant otolith formation has been reported for other species, such as red drum *Sciaenops ocellatus* (L. 1766) and coho salmon *Oncorhynchus kisutch* (Walbaum 1792) (David *et al*., 1994; Sweeting *et al*., 2004). Furthermore, Ma *et al*. (2008) suggested that the incidence of aberrant otolith formation may be an indicator of the stress experienced by fish in both hatchery and natural environments. In addition, the present study revealed that the vertebral radius of hatchery‐origin *T. rubripes* was smaller than that of wild fish. The reason for this is not known but may be related to the fact that the hatchery‐origin fish were skinnier than the wild fish. Various other abnormalities have also been reported in hatchery‐origin *T. rubripes*, including caudal fin defects (Iwamoto & Fujimoto, 1997), inter‐nostril epidermis defects (Sakurai & Hamada, 2017) and abnormal behaviour during the juvenile stage (Shimizu *et al*., 2007). Most notably, *T. rubripes* are unable to acquire tetrodotoxin under the current artificial rearing environments (Noguchi *et al*., 2006). Hence, hatchery‐origin juveniles, which are nontoxic, have higher mortality due to predation than wild juveniles, which are toxic (Shimizu *et al*., 2007). In addition to defending against predators (Itoi *et al*., 2018), tetrodotoxin is also thought to act as an immunostimulant and a stress‐relieving substance for *T. rubripes* (Amano *et al*., 2019; Honda *et al*., 2005). The amount of acquired tetrodotoxin may therefore influence the degree of fitness of individuals in natural environments. However, it has not been verified whether nontoxic hatchery‐origin fish accumulate tetrodotoxin to the same extent as wild fish after release into natural environments. Further studies on morphology, physiology and ecology should reveal more differences between wild and hatchery‐origin *T. rubripes* and help to explain why the fitness of hatchery‐origin fish is lower than that of wild fish.

### 4.5 Impact of hatchery‐release programmes

What effect do hatchery‐release programmes have on *T. rubripes* stock? For most species for which hatchery releases have been conducted, the effects on stock enhancement have not been properly quantified, and thus the effectiveness of hatchery release programmes has often been questioned and discussed (Bacon *et al*., 2015). In some cases, it has been reported that the apparent increase in stocks due to hatchery‐release programmes is actually due to climate change (Morita *et al*., 2006) or due to habitat restoration and catch limitations (Kitada *et al*., 2019). A previous study has also reported that hatchery‐origin fish contribute to an increase in stock, but concurrently suppress the increase in wild populations (Amoroso *et al*., 2017). Moreover, because hatchery‐reared fish harbour lower levels of genetic diversity than wild fish, their reproduction in the wild will reduce the genetic diversity of wild populations (Araki & Schmid, 2010). Although the reduced fitness in the wild of hatchery‐reared fish appears to be primarily caused by altered selection regimes rather than by a loss of genetic diversity, the reduction of genetic diversity of populations caused by hatchery‐release programmes will reduce the long‐term fitness of the population (Lorenzen *et al*., 2012). For example, Araki *et al*. (2009) have found that relative reproductive fitness is lower in wild‐born fish from one captive‐bred and one wild parent (far lower in those from two captive‐bred parents) than those from two wild parents for rainbow trout *Oncorhynchus mykiss* (Walbaum 1792). McGinnity *et al*. (2009) have also suggested that a continuous hatchery‐release programme for Atlantic salmon *Salmo salar* L. 1758 may disrupt the capacity of natural populations to adapt to the higher winter water temperatures associated with climate variability. These problems are more serious when hatchery‐reared juveniles are released beyond carrying capacity, resulting in the ‘replacement’ of wild fish by hatchery‐origin fish (Lorenzen *et al*., 2012). As a dramatic example, Zhivotovsky *et al*. (2012) have shown that the rapid expansion of hatchery‐release programmes for chum salmon *Oncorhynchus keta* (Walbaum 1792) has led to the replacement of wild fish by hatchery‐origin fish and the genetic swamping of a unique and rare beach‐spawning population in Lebedinoe Lake. In spite of the above examples, the hatchery‐release programmes for *T. rubripes* have continued without considering the impacts on the population, and the proportion of hatchery‐origin fish in the catch of age‐0 fish has reached 20%–30% (FRA, 2020). Continuing such a large‐scale hatchery‐release programme may harm the population sustainability (Kitada 2020) of *T. rubripes*. Based on the age and growth analysis, the present study showed that hatchery‐origin *T. rubripes* had lower fitness in natural environments throughout their lives, but it is unknown whether their offspring inherit this characteristic (lower fitness). According to Lorenzen (2005), if hatchery‐origin fish contribute to reproduction and their lower fitness is passed on to their offspring, their reduced fitness may have the strongest effect on overall population fitness and productivity. To reveal the impact of hatchery releases on the long‐term fitness of populations or species, it is necessary to investigate the relative reproductive success of hatchery‐origin *T. rubripes*, the fitness of their offspring and their impact on genetic diversity.

## 5 CONCLUSION

The present study successfully estimated the age of *T. rubripes* using otolith sections and revealed that these estimates were more accurate than those based on vertebrae, commonly used in pufferfish. This result strongly supports that otoliths, rather than conventional vertebrae, should be used to estimate age and growth for important fishery stocks, such as *T. rubripes*. This will enable more accurate future stock assessments and management, and can be used to estimate hatchery‐release effects.

The increased accuracy of age estimation clarified that the fitness (growth, condition and mortality) of hatchery‐reared *T. rubripes* is lower than that of wild fish throughout their lifetime. In addition, for species with limited nursery grounds, such as *T. rubripes*, the probability that hatchery‐reared juveniles will partially replace wild juveniles, rather than be added to the stock, suggests that hatchery‐release programmes may in fact be having a negative impact on the stock. Moreover, the contribution of hatchery‐origin fish to reproduction will reduce the genetic diversity of the population and their lower fitness may be passed on to offspring, leading to a reduction in the long‐term fitness of the *T. rubripes* population. This may be one factor responsible for the long‐term failure of tiger pufferfish reproduction, which is currently being experienced (FRA, 2020). Therefore, future studies should focus on the interaction between hatchery‐reared and wild fish in natural environments, differences in the behavioural migration ecology of hatchery‐reared fish over their lifetime and the genetic impacts of the hatchery‐release programme for *T. rubripes*. The results of this study strongly suggest that it should be reconsider the implementation of the current *T. rubripes* hatchery‐release programmes, including methods such as the fin clipping. In addition, rather than focusing solely on hatchery‐release programmes, policies should also be implemented to increase the reproductive success of wild populations, such as habitat protection.

## AUTHOR CONTRIBUTIONS

Y.O. contributed to laboratory work, data analysis and manuscript preparation. A.Y. contributed to study design, field work, data analysis, manuscript preparation and project funding.

## FUNDING INFORMATION

Nagasaki University Priority Research Subject Project Based on Medium‐term Goals and Plans.

## Supporting information


**Appendix S1** Supporting Information
