# Supplementary material for: Reduced lifetime fitness (growth, body condition and survivability) of hatchery‐reared tiger pufferfish Takifugu rubripes compared to wild counterparts
Source: J Fish Biol. 2022 Sep 7;101(5):1270–84. doi: 10.1111/jfb.15199 (PMC9826526; doi:10.1111/jfb.15199)
Supplement: Supplementary file 1 — Appendix S1 Supporting Information [file JFB-101-1270-s001.pptx]

## Slide 1
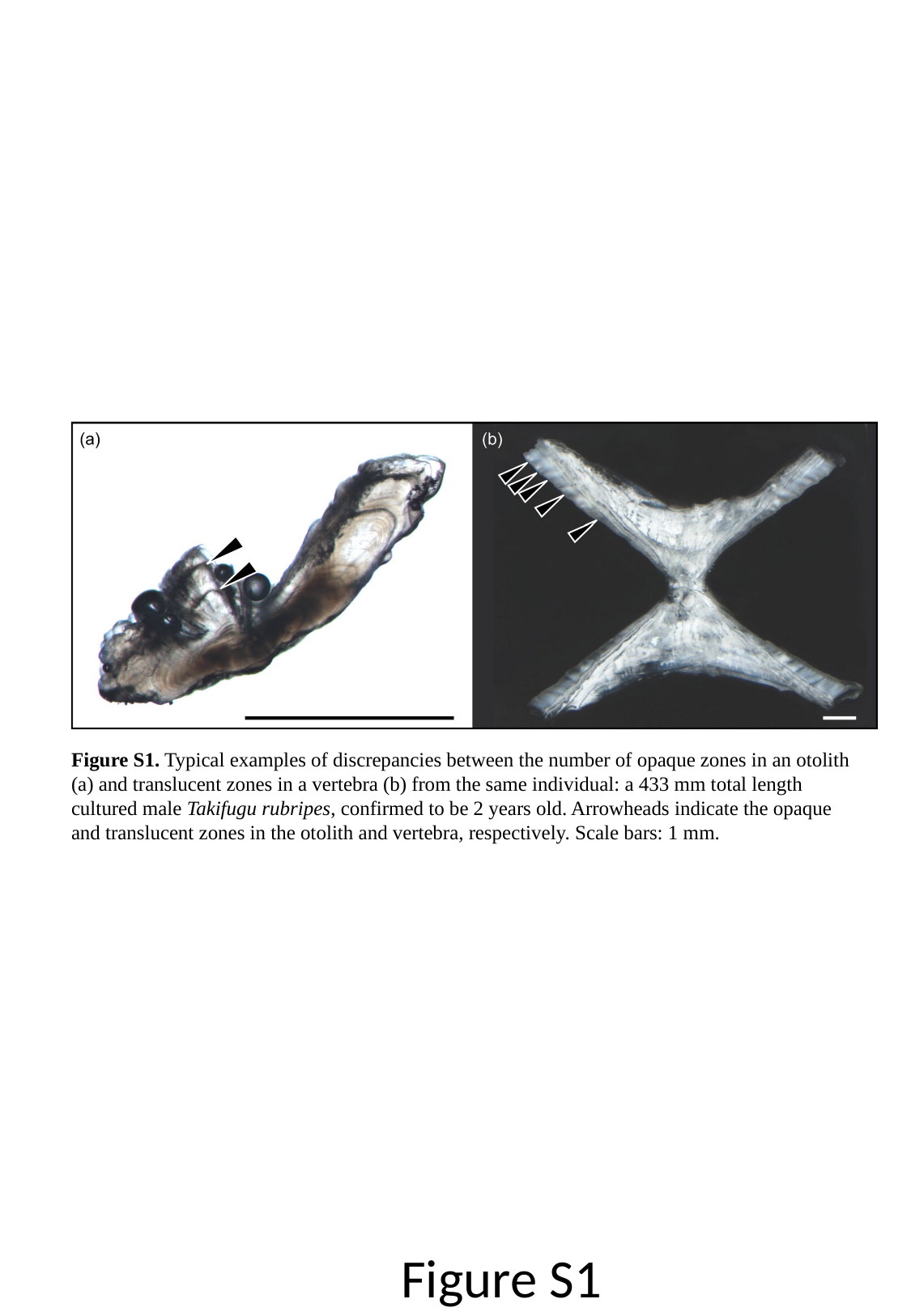

Figure S1. Typical examples of discrepancies between the number of opaque zones in an otolith (a) and translucent zones in a vertebra (b) from the same individual: a 433 mm total length cultured male Takifugu rubripes, confirmed to be 2 years old. Arrowheads indicate the opaque and translucent zones in the otolith and vertebra, respectively. Scale bars: 1 mm.
Figure S1

## Slide 2
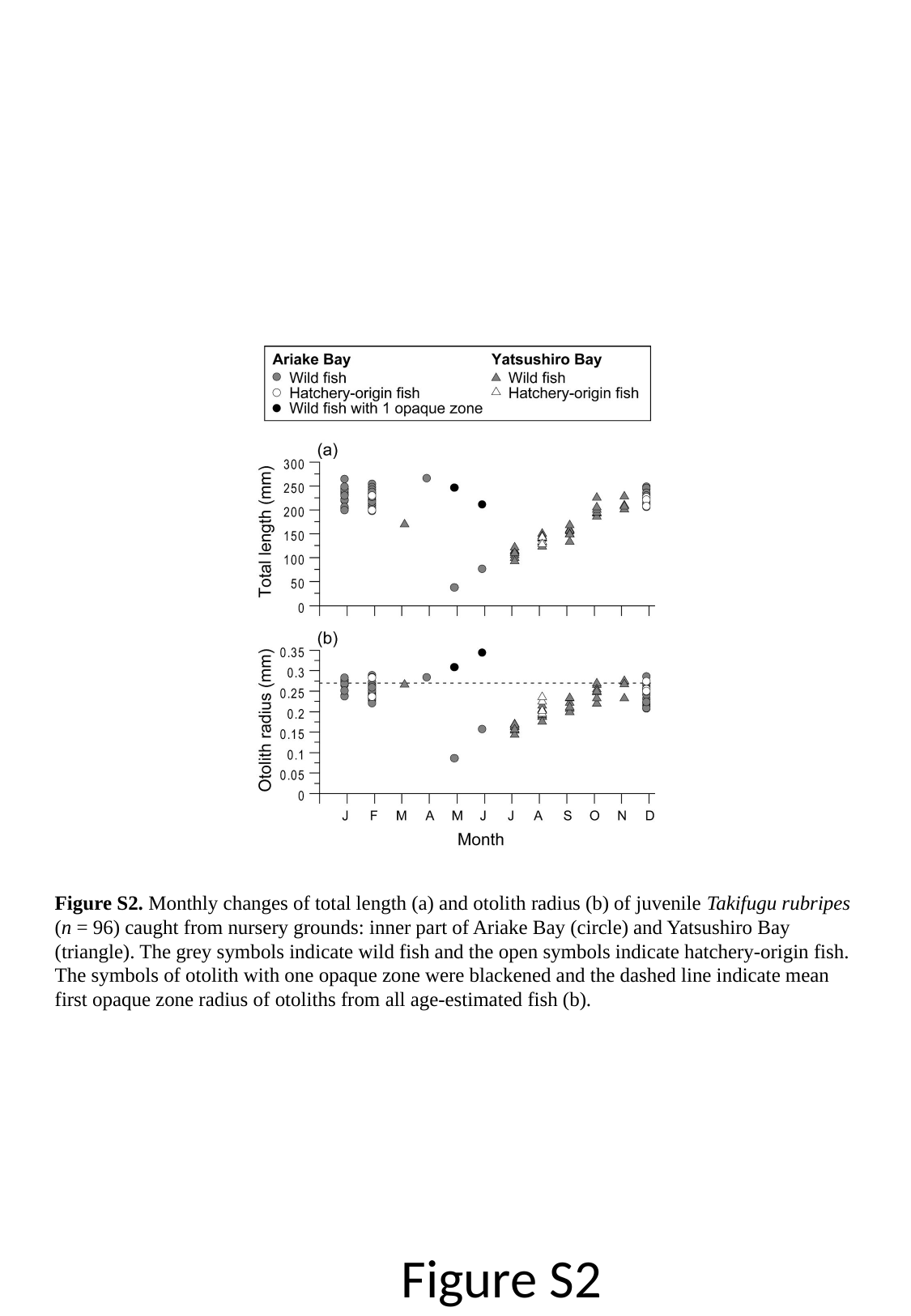

Figure S2. Monthly changes of total length (a) and otolith radius (b) of juvenile Takifugu rubripes (n = 96) caught from nursery grounds: inner part of Ariake Bay (circle) and Yatsushiro Bay (triangle). The grey symbols indicate wild fish and the open symbols indicate hatchery-origin fish. The symbols of otolith with one opaque zone were blackened and the dashed line indicate mean first opaque zone radius of otoliths from all age-estimated fish (b).
Figure S2

## Slide 3
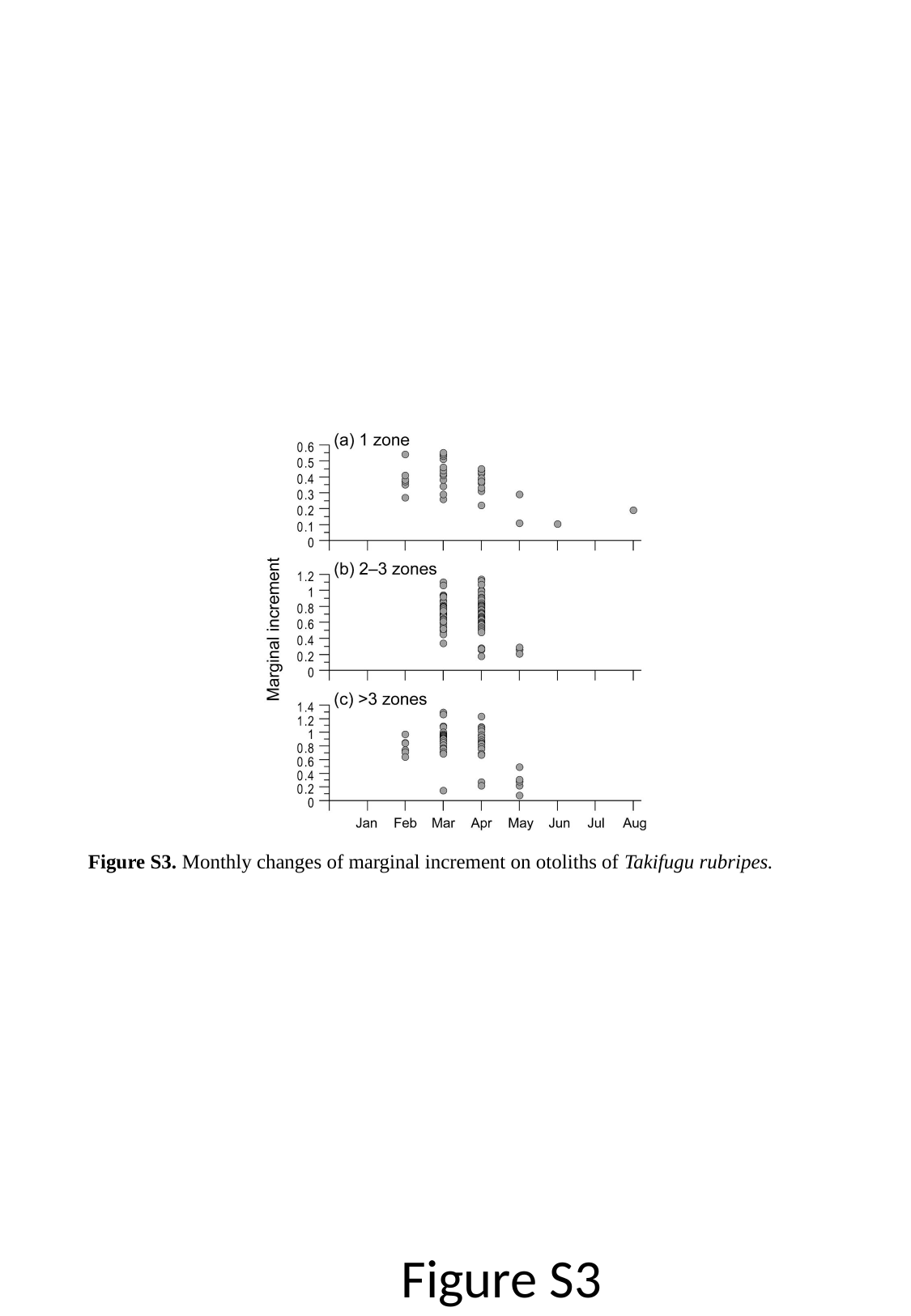

Figure S3. Monthly changes of marginal increment on otoliths of Takifugu rubripes.
Figure S3

## Slide 4
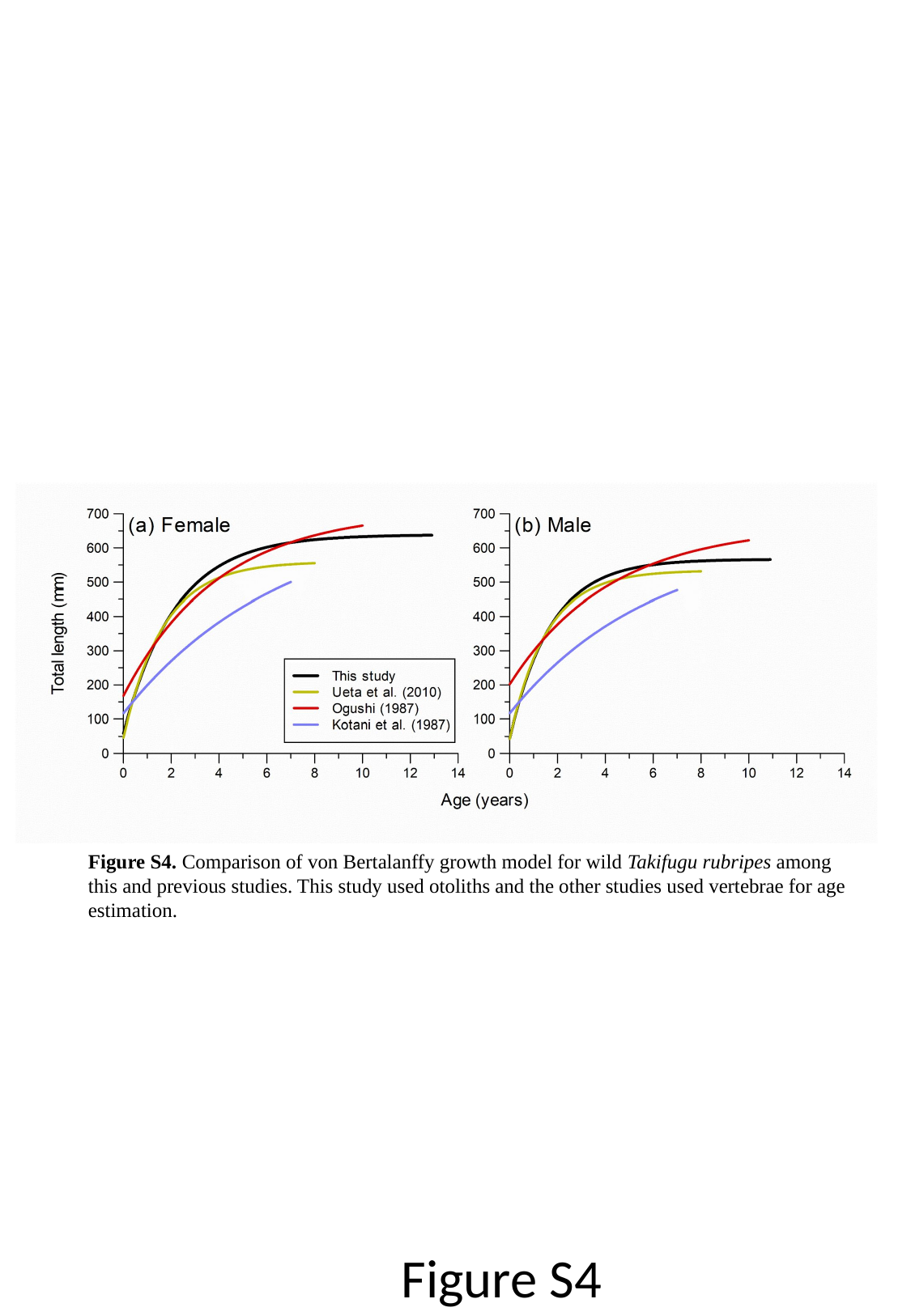

Figure S4. Comparison of von Bertalanffy growth model for wild Takifugu rubripes among this and previous studies. This study used otoliths and the other studies used vertebrae for age estimation.
Figure S4

## Slide 5
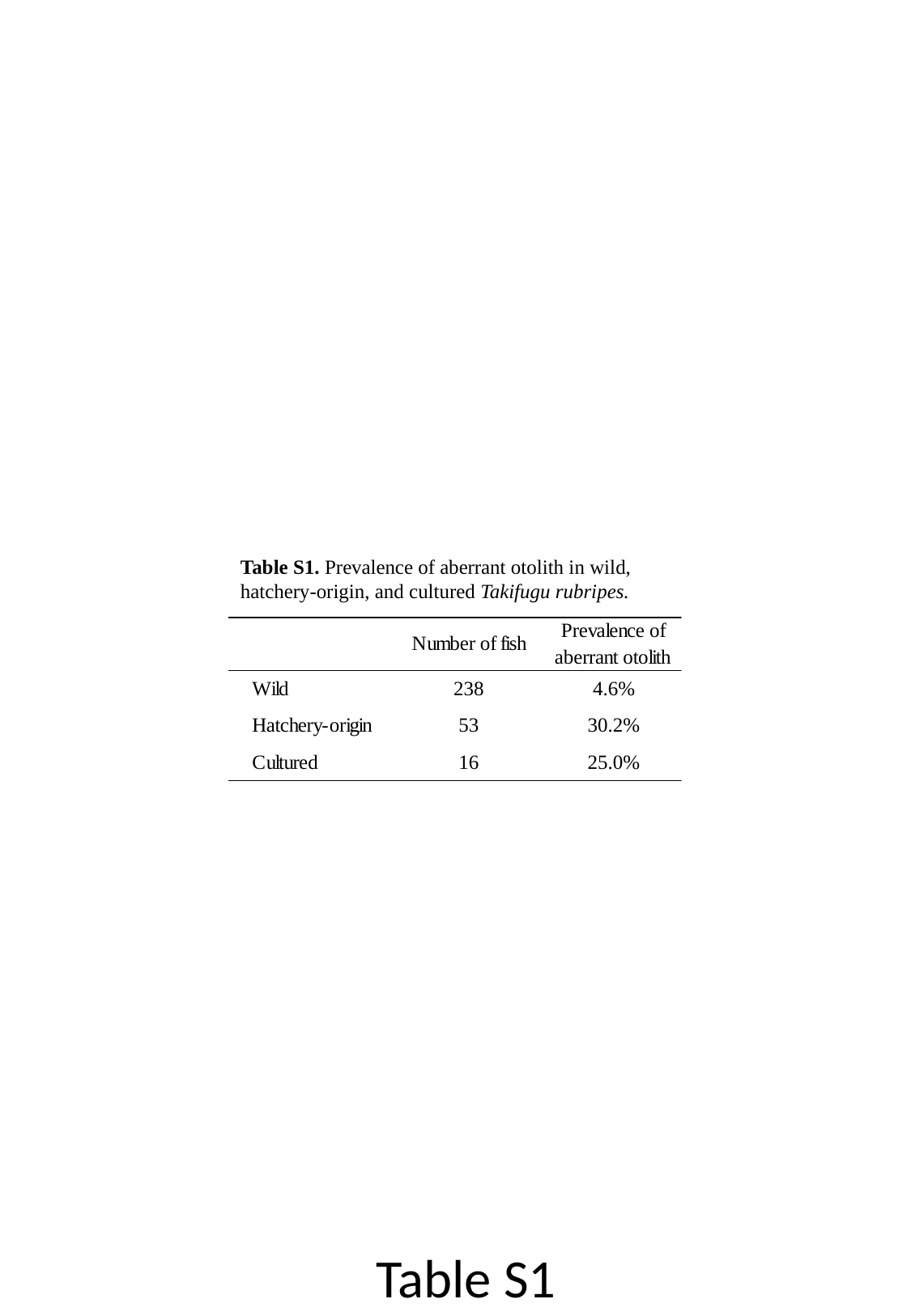

Table S1. Prevalence of aberrant otolith in wild, hatchery-origin, and cultured Takifugu rubripes.
Table S1

## Slide 6
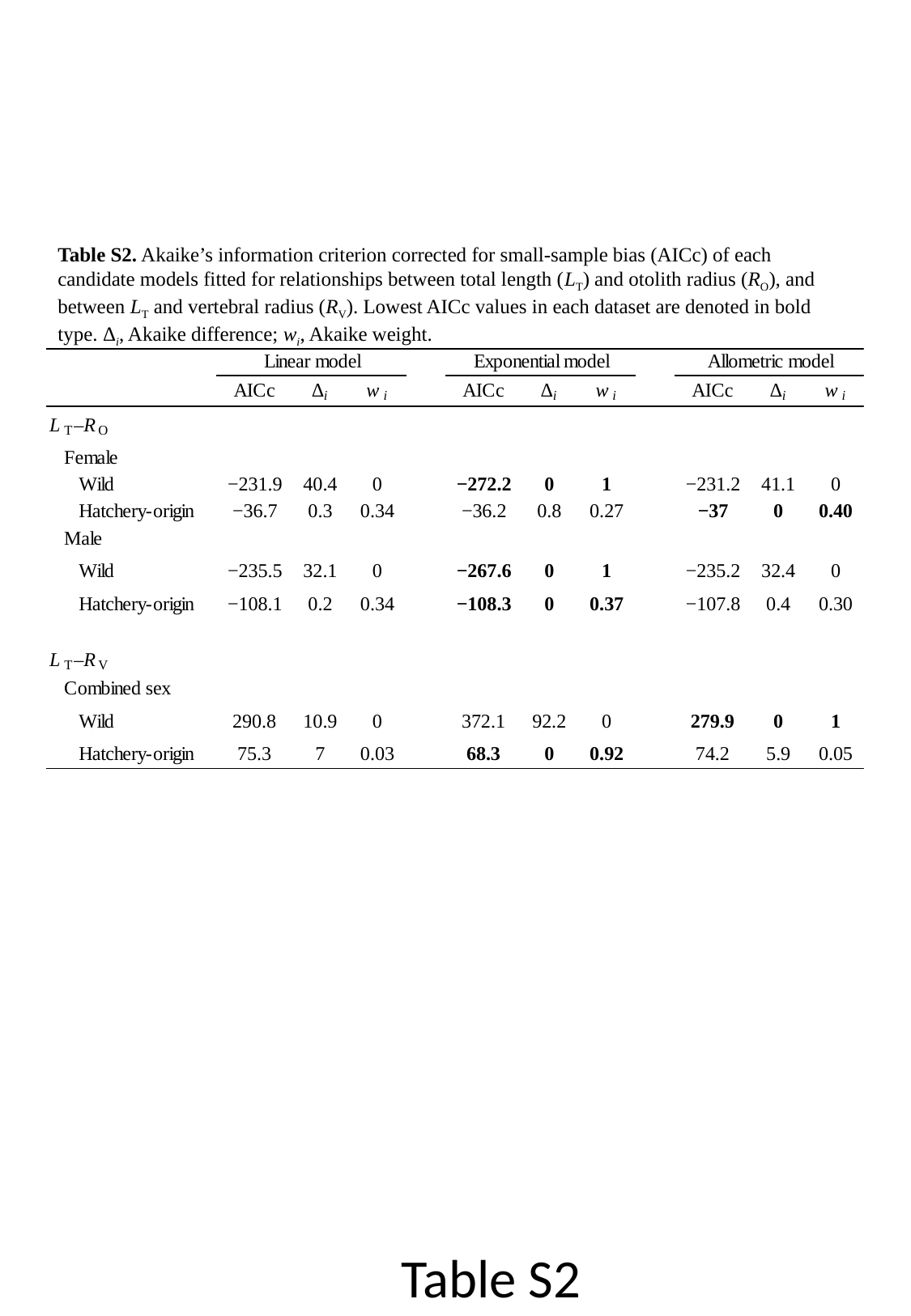

Table S2. Akaike’s information criterion corrected for small-sample bias (AICc) of each candidate models fitted for relationships between total length (LT) and otolith radius (RO), and between LT and vertebral radius (RV). Lowest AICc values in each dataset are denoted in bold type. Δi, Akaike difference; wi, Akaike weight.
Table S2

## Slide 7
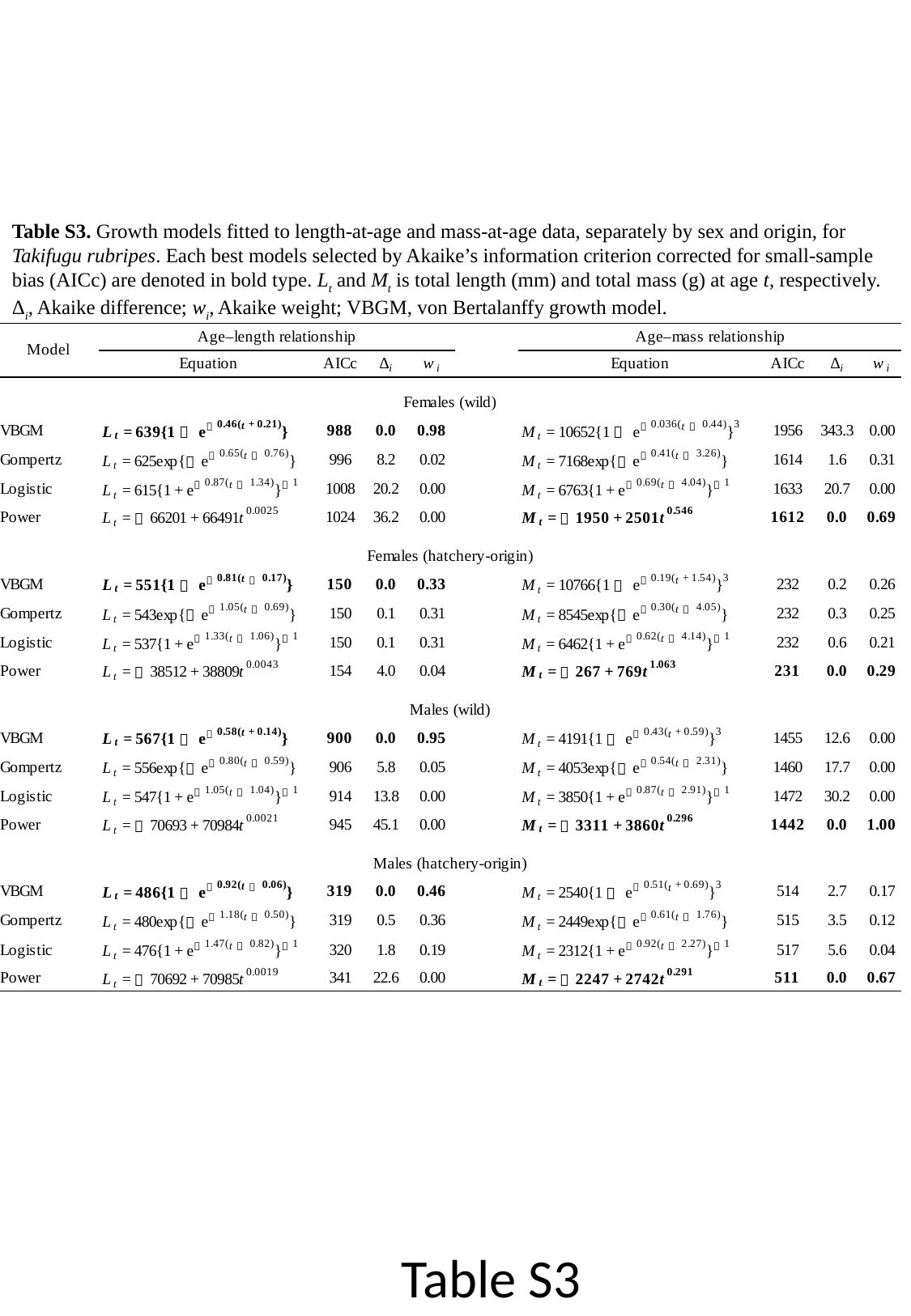

Table S3. Growth models fitted to length-at-age and mass-at-age data, separately by sex and origin, for Takifugu rubripes. Each best models selected by Akaike’s information criterion corrected for small-sample bias (AICc) are denoted in bold type. Lt and Mt is total length (mm) and total mass (g) at age t, respectively. Δi, Akaike difference; wi, Akaike weight; VBGM, von Bertalanffy growth model.
Table S3

## Slide 8
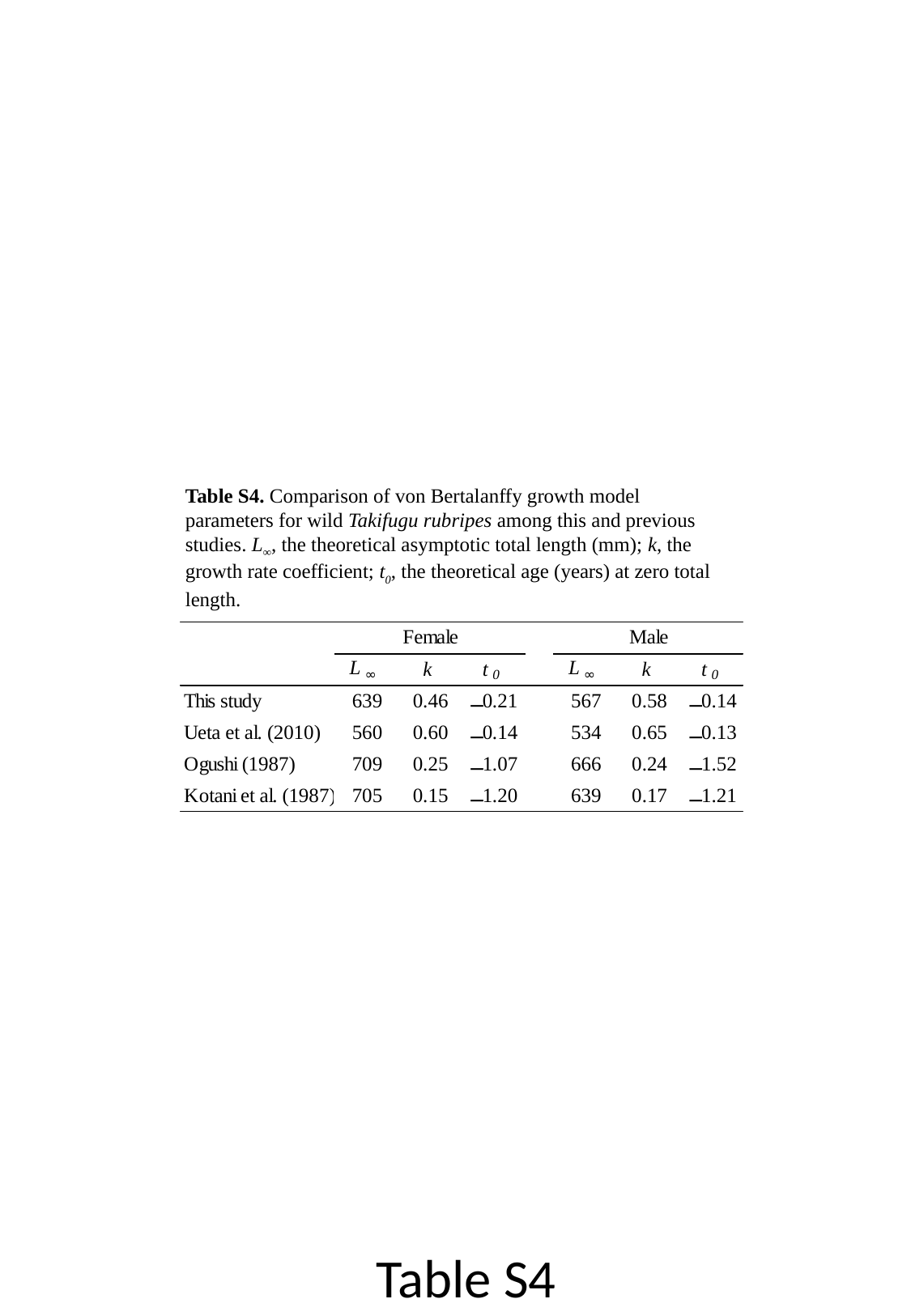

Table S4. Comparison of von Bertalanffy growth model parameters for wild Takifugu rubripes among this and previous studies. L∞, the theoretical asymptotic total length (mm); k, the growth rate coefficient; t0, the theoretical age (years) at zero total length.
Table S4
